# Functional Importance Backbones of the Brain at Rest, Wakefulness, and Sleep

**DOI:** 10.3390/brainsci15070772

**Published:** 2025-07-20

**Authors:** Klaus Lehnertz, Timo Bröhl

**Affiliations:** 1 Department of Epileptology, University of Bonn Medical Centre, Venusberg Campus 1, 53127 Bonn, Germany; timo.broehl@uni-bonn.de; 2 Helmholtz-Institute for Radiation and Nuclear Physics, University of Bonn, Nussallee 14–16, 53115 Bonn, Germany; 3 Interdisciplinary Center for Complex Systems, University of Bonn, Brühler Straße 7, 53175 Bonn, Germany

**Keywords:** brain dynamics, EEG, centrality, functional brain network, time dependence, network physiology

## Abstract

**Background**: The brain is never truly at rest. Even in the absence of external tasks, it remains active, continuously organizing itself into large-scale resting-state networks involved in shaping our internal thoughts and experiences. Understanding the networks’ structure and dynamics is key to uncovering how the brain functions as a whole. While previous studies have mapped resting-state networks and explored the roles of individual brain regions (network vertices), the relevance of the time-dependent functional interactions (network edges) between them remains largely unexplored. **Methods**: Here, we assess this relevance by elucidating the time-evolving importance of both brain regions and their interactions, associated with the networks’ constituents, using the fundamental concept of centrality. We investigate long-term electrophysiological recordings of brain dynamics from more than 100 participants and reveal new insights into how resting-state networks are organized over longer times. **Results**: Our findings reveal that the functional architecture of brain networks in a resting state is critically shaped by the dynamic interplay between brain regions. We identified functional importance backbones–core sets of dynamically central vertices and edges–whose configuration varies significantly between subgroups and further varies with different brain states, including wakefulness and sleep. Notably, regions associated with the default mode network exhibited adaptable patterns of centrality, challenging the notion of static network cores. **Conclusions**: By considering the temporal evolution of both vertices and edges, we provide a more comprehensive understanding of intrinsic brain activity and its functional relevance. This dynamic perspective reveals how the brain’s intrinsic activity is coordinated across space and time, highlighting the existence of functional importance backbones that adapt to different brain states.

## 1. Introduction

That the brain is not idle when at rest is known since early observations of cerebral metabolism [1] and Hans Berger’s works on the human electroencephalogram [2]. Spontaneous (or resting-state) brain activity persists in the absence of external task direction. Functional imaging of the brain’s resting-state activity–e.g., using positron emission tomography, functional magnetic resonance imaging, or functional near-infrared spectroscopy–provided evidence for specific functional resting-state (or task-negative) networks. A resting-state network is assumed to comprise a group of brain structures with statistical relationships between signals obtained from small volumes of brain tissue from the group being stronger than relationships between signals from volumes not involved in the group. Brain networks at rest comprise somato-motor, visual occipital and auditory temporal, and several associative networks covering fronto-temporal-parietal cortices (dorsal attention, default, language, and control) [3,4,5,6,7,8,9]. Identifying and studying these intrinsic and spontaneous networks is important, since they can provide insight into the functional architecture of the brain. The most prominent and largest resting-state network–the default mode network (DMN) or medial fronto-parietal network–is composed of a few distributed brain areas that are functionally de-activated during constrained, task/goal-directed behavior. The ventral precuneus and the ventral posterior cingulate cortex, the lateral posterior parietal cortex, the medial prefrontal cortex and the lateral temporal cortex comprise this network [3,9,10,11,12], which accounts for a large fraction of the brain’s anatomy.

The organization of resting-state networks has also been investigated using noninvasive electrophysiological recording techniques such as electroencephalography (EEG) [13,14,15,16,17,18,19,20] and magnetoencephalography (MEG) [21,22,23,24] as well as invasive techniques such as intracranial EEG [25,26]. With high temporal resolution and whole-head coverage, particularly the noninvasive techniques provide a unique window into the dynamics of large-scale brain networks. Interestingly, previous research reported on only some degree of correspondence between resting-state networks observed with functional imaging and electrophysiological techniques. Incongruences are often attributed to methodological aspects, differences in analysis techniques, and to the nonstationarity of brain dynamics and resting-state networks [27,28,29,30,31,32,33]. Identifying most important or central constituents of brain-wide networks can help to improve their characterization and may facilitate a comparison between findings from different recording modalities. This can be achieved with one of the most fundamental concepts in network science, namely centrality [34], that details the role of a network constituent for structure and dynamics. Since the notion of “importance” is not unambiguous, central constituents can be identified with various centrality metrics, based on different conceptual approaches. None of these metrics is superior to the others as every metric is appropriate for some but not all facets of a constituent’s importance in the larger network. Some previous studies made use of centrality concepts to characterize resting-state networks [35,36,37,38], but these studies largely focused on network vertices using a few metrics only [39]. From the general point of view of centrality, the question arises whether this suffices to adequately characterize brain-wide networks. More importantly, little is known about the importance of edges in these networks.

To characterize the brain-wide importance of vertices and edges of networks, we developed a new analysis approach for investigating large-scale time-evolving functional brain networks [40,41] that overcomes current methodological limitations. We hypothesized that time-evolving functional brain networks from different subjects exhibit spatial and temporal commonalities that reflect characteristics associated with a potential functional importance backbone (FIB) of the brain. To this end, we applied our approach that is based on centrality concepts jointly defined for vertices and edges [42,43] to EEG recordings of more than 100 human subjects.

## 2. Materials and Methods

### 2.1. Data

We investigated EEG data acquired from 111 subjects (57 females, 54 males; age range 19–81 yrs., median 33 yrs.) without and with central nervous system (CNS) diseases (76 subjects with either focal or genetic epilepsy). All subjects participated in earlier studies [44,45,46,47,48] and signed informed consent that their data could be used and published for research purposes after being provided with written information and being given the opportunity to ask further questions. The studies were approved by the ethics committee of the University of Bonn and were conducted adhering to the principles outlined in the Declaration of Helsinki.

All subjects underwent a continuous, task-free 1-h EEG recording, and for subjects that received CNS medication, this was kept stable at least 24 h before the recording. EEG data were recorded during the early afternoon (2 p.m.–3 p.m.) to minimize possible influences from biological rhythms. [49]. We also examined continuous, long-term EEG recordings from nine subjects, admitted for evaluation of epilepsy risk, that lasted several days.

EEG data were recorded with V=19 electrodes placed according to the 10–20 system [50], and Cz served as physical reference. Table 1 summarizes which electrodes sample which brain regions [51,52]. Data were sampled at 256 Hz using a 16 bit analogue-to-digital converter (Micromed, S.p.A., Mogliano Veneto, Italy) and were band-pass filtered offline between 1–45 Hz (4th order Butterworth characteristic). A notch filter (3rd order) was used to suppress contributions at the line frequency (50 Hz). We visually inspected all recordings for strong artifacts (e.g., subject movements or amplifier saturation) and excluded such data from further analyses.

### 2.2. From EEG Data to Time-Evolving Functional Brain Networks

We derived temporal sequences of undirected, weighted [53] and fully connected functional brain networks by associating network vertices with brain regions sampled by the EEG electrodes and edge weights with the time-varying strength of interaction between pairs of vertices ((n,m)=1,…,V;n≠m). Given the important role synchronization plays in brain functioning [54,55,56,57,58], we estimate the time-varying strength of interaction by calculating the mean phase coherence [59] $R_{nm}$ employing a sliding-window approach [30,60]:

$$R_{nm}=\left|\frac{1}{T}\sum_{j=0}^{T-1}e^{i\;\left(\Phi_{n}\left(j\right)-\Phi_{m}\left(j\right)\right)}\right|.$$

*T* is the number of data points per window (T=4096; window duration: 20.48 s; non-overlapping windows) and $\Phi_{n}$ is the instantaneous phase time series of vertex *n* that we derived from the Hilbert transform of the EEG time series of that vertex. $R_{nm}$ is confined to the range [0,1], where $R_{nm}=1$ indicates fully phase-synchronized brain regions, while $R_{nm}=0$ indicates no phase synchronization.

Note that with this analytic signal approach the instantaneous frequency relates to the predominant frequency in the Fourier spectrum (particularly in case of two or more superimposed oscillatory components) [61]. In an EEG time series, the predominant frequency may be subject to fluctuations which results in an instantaneous frequency that varies rhythmically around the predominant frequency and thus to spurious estimates of the instantaneous phase. Such effects can be reduced, e.g., by taking the temporal average.

We also note that it might be more reasonable–from an electrophysiological point of view–to investigate adaptively interactions between predominant EEG rhythms (e.g., via the Hilbert transform) than to look at interactions in some a priori fixed frequency bands (e.g., via wavelet transform) for which there is no power in the time series [62].

### 2.3. Estimating Vertex and Edge Centralities

Centrality is one of the most fundamental concepts in network science [63,64,65,66,67], and the various centrality metrics assess the different roles network constituents (vertices and edges) play in a network. Here, we make use of centrality concepts jointly defined for vertices and edges that can be classified as path-based concepts (betweenness centrality $C^{B}$ and closeness centrality $C^{C}$) or strength-based concepts (eigenvector centrality $C^{C}$, strength centrality $C^{S}$, and nearest neighbor centrality $C^{N}$).

The functional brain networks investigated here are weighted, undirected and fully connected networks that consist of sets of vertices V and edges E, with $V=\left|V\right|$ and $E=\left|E\right|$ denoting the number of vertices and edges, respectively. We do not consider self-loops or parallel edges.

*Path-based centrality concepts and metrics*

Centrality concepts that are based on shortest paths require the definition of “length” $d_{ij}$ of a path between vertices *i* and *j*. Since an edge weight represents the strength of an interaction between two vertices, we consider a path to be shorter the stronger the interaction along this path is. Consequently, we relate $d_{ij}$ of path P between vertices *i* and *j* to the sum of the inverse weights of edges along this path [65]. A shortest path can be defined as the path between two vertices for which the sum of the inverse weights of edges along this path is minimal [42].

Betweenness centrality $C^{B}$ rates a network constituent the more central the more shortest paths pass through this constituent. Betweenness centrality of vertex/edge *k* can be defined as [68,69,70]

(1) $$C_{v,e}^{B}\left(k\right)=\frac{2}{F}\sum_{i\neq j}\frac{q_{ij}\left(k\right)}{G_{ij}},$$

where $k\in \left\{1,\ldots ,V\right\}$, resp. $k\in \left\{1,\ldots ,E\right\}$, $\left\{i,j\right\}\in \left\{1,\ldots ,V\right\}$, $q_{ij}\left(k\right)$ is the number of shortest paths between vertices *i* and *j* running through vertex/edge *k*, and $G_{ij}$ is the total number of shortest paths between vertices *i* and *j*. The normalization factor is F=(V−1)(V−2) in case of vertices and F=V(V−1) in case of edges.

Closeness centrality $C^{C}$ rates a constituent the more central the shorter the paths that connect this constituent to every other reachable constituent of the same type. Closeness centrality of vertex *k* is defined as [63]:

(2) $$C_{v}^{C}\left(k\right)=\frac{V-1}{\sum_{i}d_{ik}},$$

with $\left\{k,i\right\}\in \left\{1,\ldots ,V\right\}$.

Closeness centrality of edge *k* between vertices *a* and *b* can be defined as [42]:

(3) $$\begin{array}{cc} C_{e}^{C}\left(k\right) & =\frac{E-1}{\sum_{i}\left(d_{ia}+d_{ib}\right)}=\frac{E-1}{\frac{1}{C_{v}^{C}\left(a\right)}+\frac{1}{C_{v}^{C}\left(b\right)}}\\ \; & =\left(E-1\right)\frac{C_{v}^{C}\left(a\right)\;C_{v}^{C}\left(b\right)}{C_{v}^{C}\left(a\right)+C_{v}^{C}\left(b\right)}, \end{array}$$

with $k\in \left\{1,\ldots ,E\right\}$ and $\left\{a,b,i\right\}\in \left\{1,\ldots ,V\right\}$.

*Strength-based centrality concepts and metrics*

Eigenvector centrality $C^{E}$ rates a network constituent central if its adjacent constituents of the same type are also central. Eigenvector centrality of vertex [71] or edge [42] *k* is defined as the *k*th entry of the eigenvector $\vec{v}$ corresponding to the dominant eigenvalue $\lambda_{\max}$ of matrix M, which can be derived from the eigenvector equation $M\vec{v}=\lambda \vec{v}$ using the power iteration method:

(4) $$C_{v,e}^{E}\left(k\right)=\frac{1}{\lambda_{\max}}\sum_{i}M_{ki}\;C_{v,e}^{E}\left(i\right).$$

In case of vertices, $\left\{k,i\right\}\in \left\{1,\ldots ,V\right\}$ and M denotes the weight matrix $W^{\left(v\right)}\in R_{+}^{V\times V}$, with $W_{ki}^{\left(v\right)}$ denoting the weight of the edge between vertices *k* and *i*. In case of edges, $\left\{k,i\right\}\in \left\{1,\ldots ,E\right\}$ and M denotes the weight matrix $W^{\left(e\right)}\in R_{+}^{E\times E}$ whose entries $W_{ki}^{\left(e\right)}$ are assigned the average weight of edges *k* and *i* if these edges are connected to a same vertex, and 0 otherwise.

Strength centrality $C^{S}$ rates a vertex the more central the stronger its interactions with adjacent vertices are. Strength centrality of vertex *k* is defined as:

(5) $$C_{v}^{S}\left(k\right)=\sum_{i}W_{ki}^{\left(v\right)},$$

with $\left\{k,i\right\}\in \left\{1,\ldots ,V\right\}$ and the weight matrix element $W_{ki}^{\left(v\right)}$ denoting the weight of the edge between vertices *k* and *i*.

Nearest-neighbor centrality $C^{N}$ is a concept for an edge centrality analogous to strength centrality (or vertex strength) [43]. It rates an edge the more central the larger its weight and the more similar and the higher the strengths of the vertices which are connected by that edge. Nearest-neighbor centrality of edge *k* between vertices *a* and *b* can be defined as [43]:

(6) $$C_{e}^{N}\left(k\right)=\frac{C_{v}^{S}\left(a\right)+C_{v}^{S}\left(b\right)-2W_{k}}{|C_{v}^{S}\left(a\right)-C_{v}^{S}\left(b\right)|+1}\;W_{k},$$

where $k\in \left\{1,\ldots ,E\right\}$ and $\left\{a,b\right\}\in \left\{1,\ldots ,V\right\}$, and $W_{k}$ denotes the weight of edge *k* connecting vertices *a* and *b* (note, that an additional normalization factor $\left(\frac{1}{2(V-2)}\right)$ can be considered when aiming at a comparison with other edge centrality concepts, since established edge centralities (e.g., $C_{e}^{B}$) are also normalized with respect to the total number of vertices).

Data analysis was performed via Python 3.8.

### 2.4. Statistical Analyses

We employed the Mann-Whitney-U-Test (p<0.05, after Bonferroni correction) to evaluate differences between FIB (group means of centrality values of all individual constituents) from different subgroups of subjects.

## 3. Results

### 3.1. Identifying Functional Importance Backbones (FIBs) of the Brain

**Figure 1:**
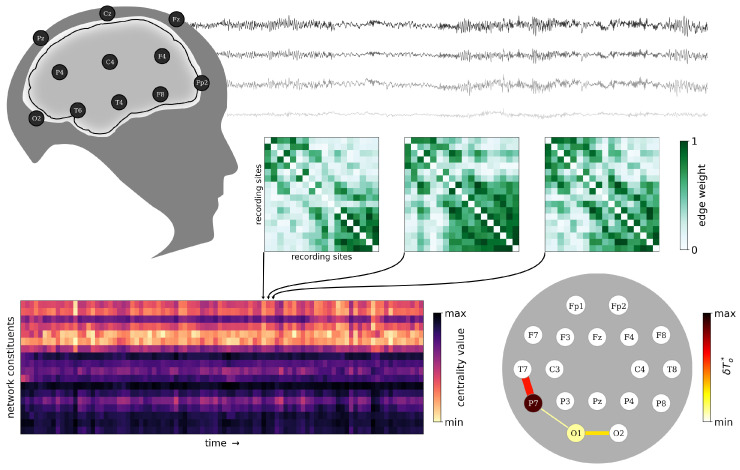
From multichannel scalp EEG recordings to the coarse-grained functional importance backbone of the brain. We performed a time-resolved synchronization-based EEG analysis to derive a temporal sequence of weighted time-evolving functional brain networks (TEFBNs; each represented as weight matrix). We identified central constituents in each TEFBN by collecting for each subject the fraction of observation time $\delta T_{o}^{★}$ during which a constituent is most central. These constituents are then projected back onto the 10–20 EEG recording layout [50] and form a characteristic functional importance backbone of the brain (FIB).

Figure 1 summarizes our analysis approach in a compact form. In a first step, we build temporal sequences of large-scale functional brain networks (time-evolving functional brain networks; TEFBNs) from the subjects’ EEG recordings utilizing a time-resolved synchronization analysis between all sampled brain regions. The latter are the networks’ vertices, and we assign the pairwise levels of synchronization to the edges’ weights. Next, we identify constituents (vertices and edges) in each brain-wide TEFBN by collecting the fraction of observation time $\delta T_{o}^{★}$ during which a constituent is most central–thereby accounting for the brain’s fluctuations in stationarity [30,47]. We refer to constituents for which $\delta T_{o}^{★}>0.05$ as important constituents and these form a characteristic functional importance backbone of the brain (FIB).

**Figure 2:**
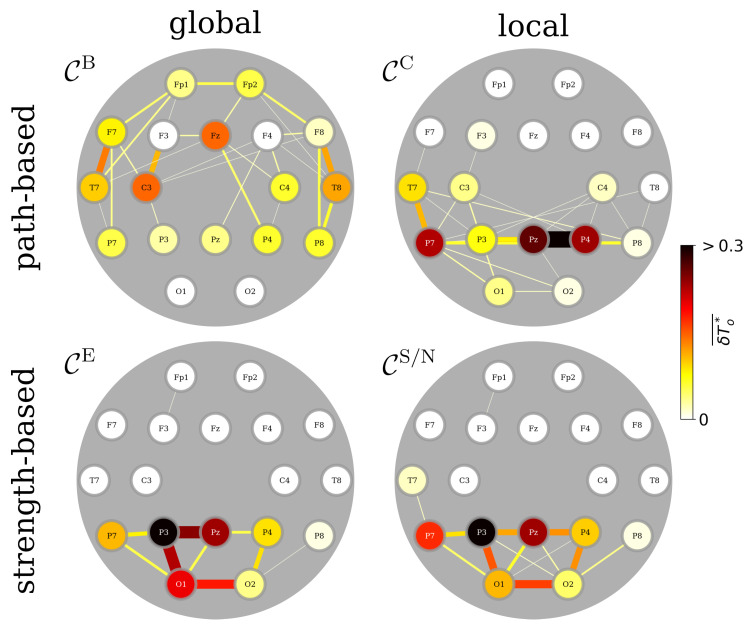
Average functional importance backbones from 111 brains. Color-coded group medians of the fraction of observation time $\delta T_{o}^{★}$ during which an individual network constituent (vertex, edge) is most central. We identified such central constituents with path-based centrality metrics (global: betweenness centrality $C^{B}$; local: closeness centrality $C^{C}$) and with strength-based centrality metrics (global: eigenvector centrality $C^{E}$; local: strength/nearest neighbor centrality $C^{S/N}$). Observation time is 1 h.

Now, depending on how we rate the constituents’ central roles in the larger network, we find various FIBs that exhibit different spatial characteristics for the relaxed and awake state (Figure 2). Concentrating on bottlenecks in a TEFBN’s path structure with betweenness centrality, this FIB mainly presents with vertices and edges highlighting fronto-temporo-central brain areas and their interactions on both hemispheres, although with a slight emphasis on the left hemisphere. A major influence on information flow between other constituents of a TEFBN can be attributed to this subnetwork. Concentrating again on a TEFBN’s path structure, we next ask with closeness centrality which constituents exert most direct influence over the other constituents. We find that the midline-right-parietal junction–formed by the influential adjacent vertices Pz and P4 together with the influential edge that connects them–as well as vertices and edges highlighting predominantly left temporo-parietal brain areas and their interactions constitute the corresponding FIB. This subnetwork controls the speed of information transfer (via short paths) to other constituents of a TEFBN. If we single out constituents that are most strongly integrated within a TEFBN (global and local strength-based centrality metrics), the respective FIBs present with vertices and edges predominantly highlighting left temporo-parietal and left parieto-occipital brain areas and their interactions. The similarity of these backbones points to a spatially tightly circumscribed FIB.

**Figure A1:**
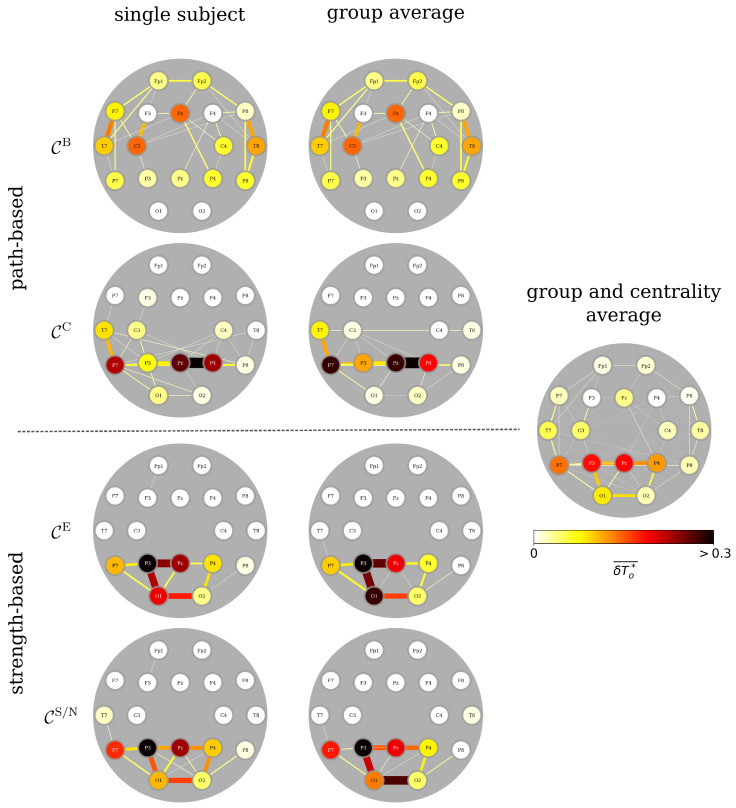
Functional importance backbones (FIBs) from a single subject (**left**) and group averages from 111 brains (**middle**). Merged FIB (**right**) is group and centrality average. Color-coded fraction of observation time $\delta T_{o}^{★}$ during which an individual network constituent (vertex, edge) is most central. We identified such central constituents with path-based centrality metrics (global: betweenness centrality $C^{B}$; local: closeness centrality $C^{C}$) and with strength-based centrality metrics (global: eigenvector centrality $C^{E}$; local: strength/nearest neighbor centrality $C^{S/N}$). These constituents were projected back onto the 10-20 EEG recording layout [50,114]. Observation time is 1 h.

Interestingly, the identified FIBs–when merged–are strongly reminiscent of the DMN, albeit in a coarse-grained resolution and projected onto the scalp (Appendix A; Figure A1). Our approach thus appears to allow to disentangle relevant cortical components of the DMN by concentrating on central constituents of brain-wide TEFBNs. In order to further substantiate this assertion, we have investigated various factors the FIBs may be sensitive to.

### 3.2. FIBs Are Insensitive to Epilepsy Types and Handedness

**Figure A2:**
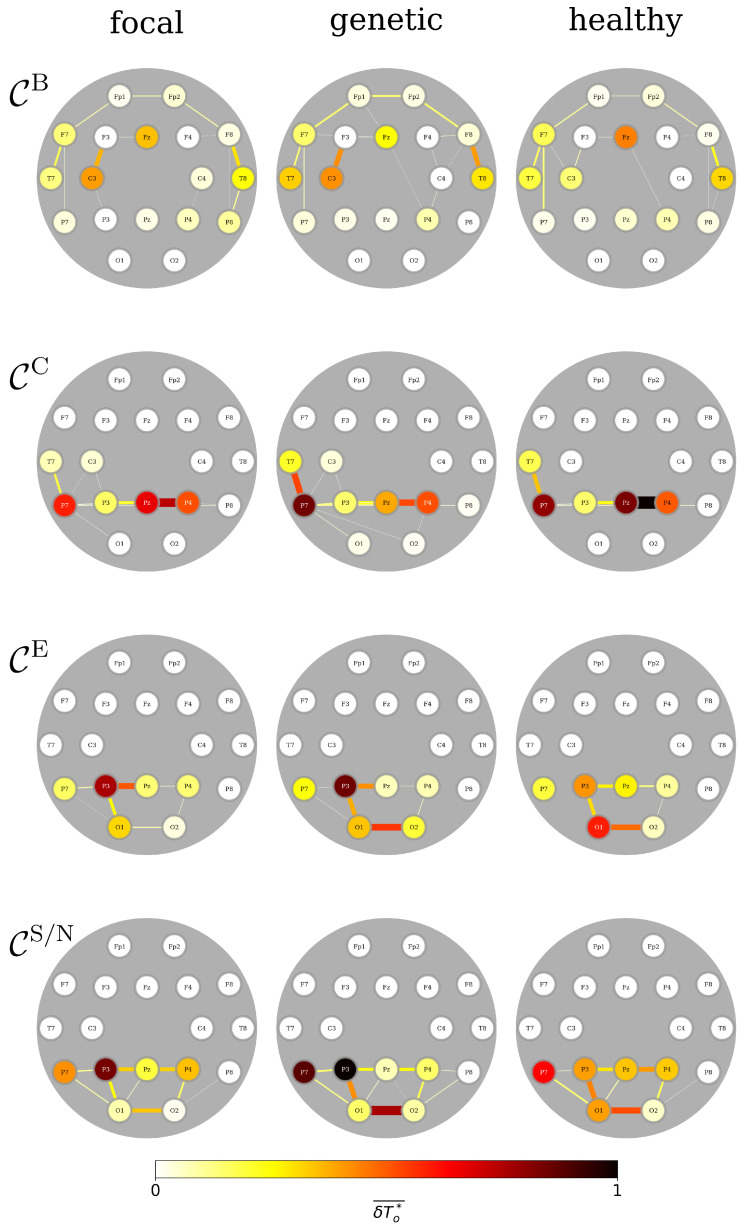
Averaged functional importance backbones (FIBs) from 61 subjects with a focal epilepsy, 15 subjects with a genetic one, and from 35 healthy subjects (without a disease of the central nervous system). There were no significant differences between FIB from the different subgroups of subjects. Abbreviations and color-coding as in Figure A1.

To begin with, and taking into account a large number of observations that point to virtually all psychiatric and neurological disorders impacting on DMN function [12], we asked whether different epilepsy types present with different FIBs. Of the 111 investigated subjects, 61 had a focal epilepsy and 15 a genetic one, and there were no significant differences between the respective FIBs (Appendix A; Figure A2). Likewise, we could not identify any significant differences when comparing FIBs of subjects with epilepsy with those from the 35 subjects without a disease of the central nervous system. Our findings appear to contradict earlier, mostly fMRI-based studies on DMN dysfunction in epilepsy, which may reflect their limitations such as small sample sizes and lack of methodological standardization [72].

**Figure A3:**
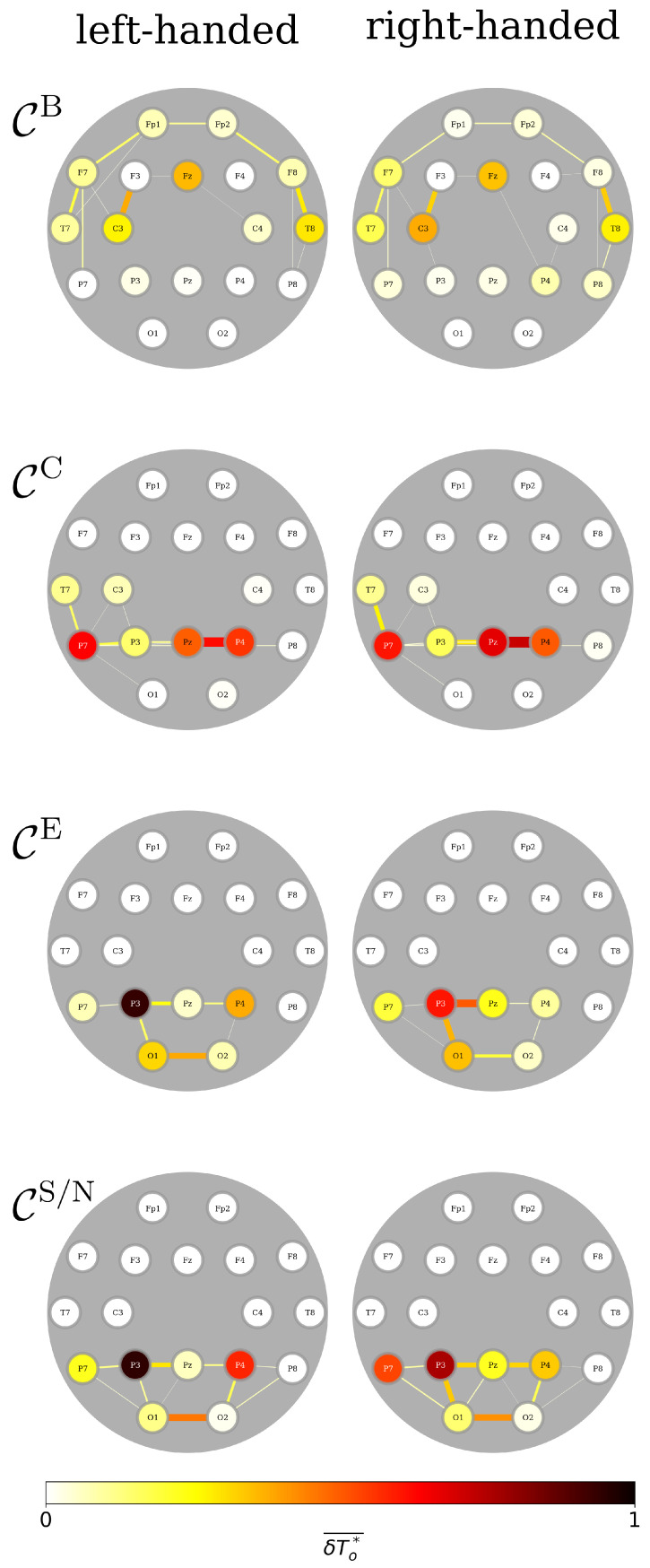
Averaged functional importance backbones (FIBs) from 14 left-handed and 97 right-handed subjects. There were no significant differences between FIB from the different subgroups of subjects. Abbreviations and color-coding as in Figure A1.

We next asked whether handedness [73] might explain the predominantly left-hemispheric representation of FIBs. Of the 111 investigated subjects, 14 demonstrated with left-handedness [74], which is usually regarded as a representative portion [75]. There were no significant differences between the respective FIBs (Appendix A; Figure A3), though we can not exclude an impact of left-hemispheric language dominance [76].

### 3.3. FIBs Are Sensitive to Sex, Age, and Continuous Attention

**Figure A4:**
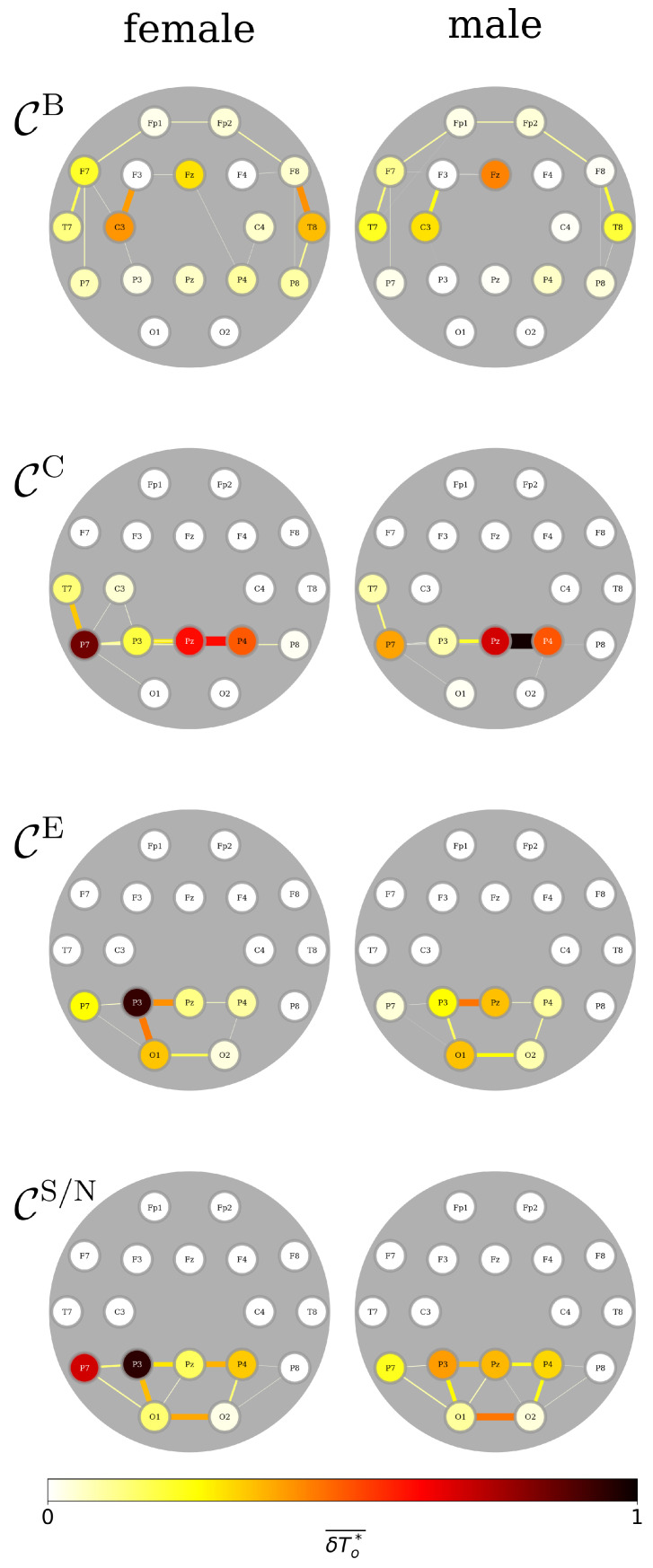
Averaged functional importance backbones (FIBs) from 57 females and 54 males. See main text for group differences. Abbreviations and color-coding as in Figure A1.

Beyond size differences, there are only few, mostly trivial differences between male and female brains [77], and where small differences were found, they were confined to the default mode network (DMN) [78]. Our findings from comparing 57 female and 54 male FIBs support this perspective (Appendix A; Figure A4). Concentrating on bottlenecks in a TEFBN’s path structure with betweenness centrality, the important role of the posterior midline cortices [79] (vertex Pz) was increased by 100 % in female brains. If we ask with closeness centrality which constituents exert most direct influence over the other constituents, we find that in male brains this characteristic was decreased for the left inferior temporal gyrus (vertex P7) by 54 % and for the edge connecting this gyrus with the left postcentral gyrus (vertices C3 and P7) by 100 %. In female brains, the important role of the midline-right-parietal edge (connecting the posterior midline cortices with the right precuneus; vertices PZ and P4) was decreased by 75 %. Testing for most strongly integrated constituents within a TEFBN, we find with eigenvector centrality the left precuneus (vertex P3) to be 73 % less integrated in female brains. With strength/nearest neighbor centrality, we do not observe significant differences between female and male brains.

**Figure A5:**
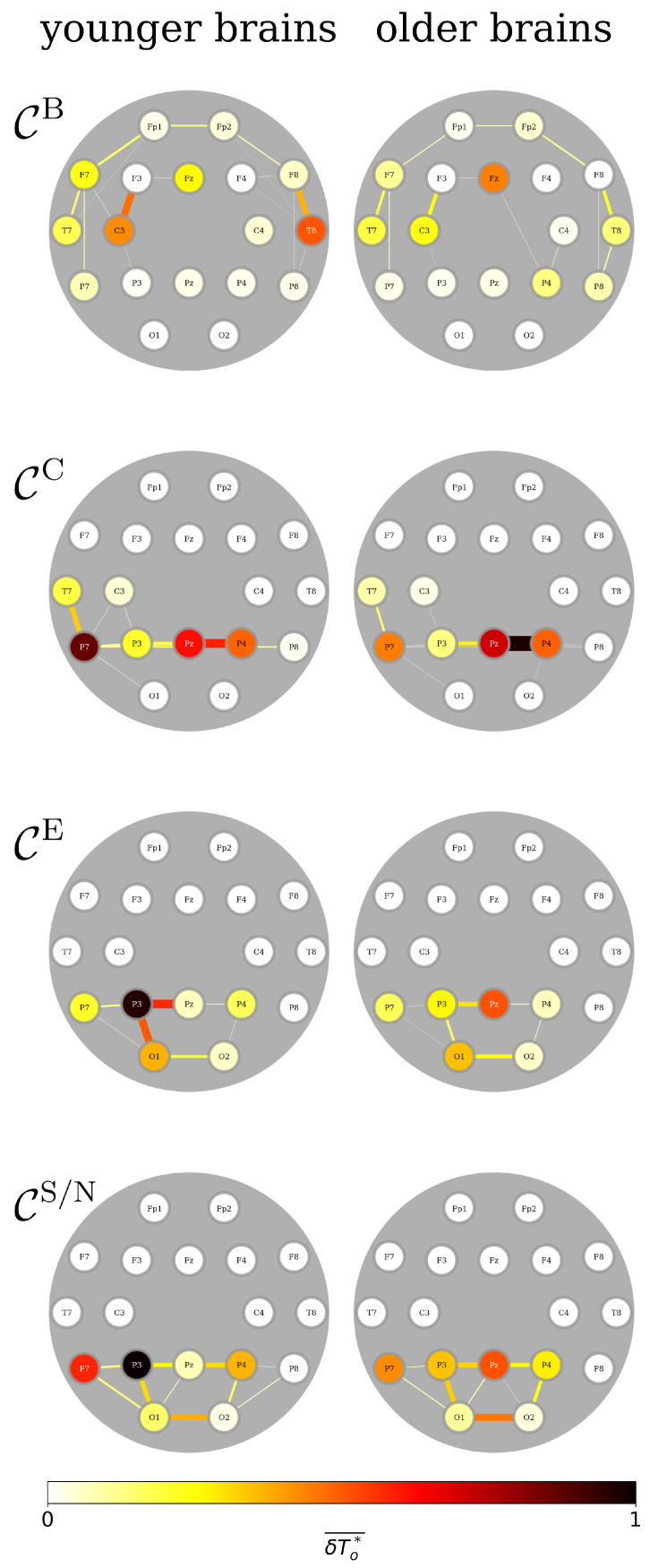
Averaged functional importance backbones (FIBs) from younger (≤33 yrs; median split) and older brains. See main text for group differences. Abbreviations and color-coding as in Figure A1.

Since age is known to influence some characteristics of functional brain networks [35,80,81,82,83], including the DMN [84], we asked whether the brains’ FIBs experience age-related alterations (Appendix A; Figure A5). Concentrating again on bottlenecks in a TEFBN’s path structure with betweenness centrality, we find the important role of the right middle temporal gyrus (vertex T8) to last about 4-fold longer in younger brains (≤33 yrs; median split), and the edge connecting that gyrus with the right middle frontal gyrus (vertices T8 and F4) lost its important role in older brains. Probing for constituents that exert most direct influence over the other constituents using closeness centrality, we identified the important role of the left inferior temporal gyrus (vertex P7) to last about 2-fold longer in younger than in older brains. Testing for most strongly integrated constituents within a TEFBN, we find in younger brains with both global and local strength-based centrality metrics the important role of the left precuneus to last 3-to-4-fold longer (vertex P3) and of the left inferior temporal gyrus (vertex P7) to last about 2-fold shorter than in older brains. The important role (assessed with nearest neighbor centrality) of the edge connecting the aforementioned brain regions (vertices P7 and P3) lasted 3-to-4-fold longer in younger brains, and the edge connecting the right inferior temporal and the right middle occipital gyrus (vertices P8 and O2) lost its important role in older brains.

**Figure A6:**
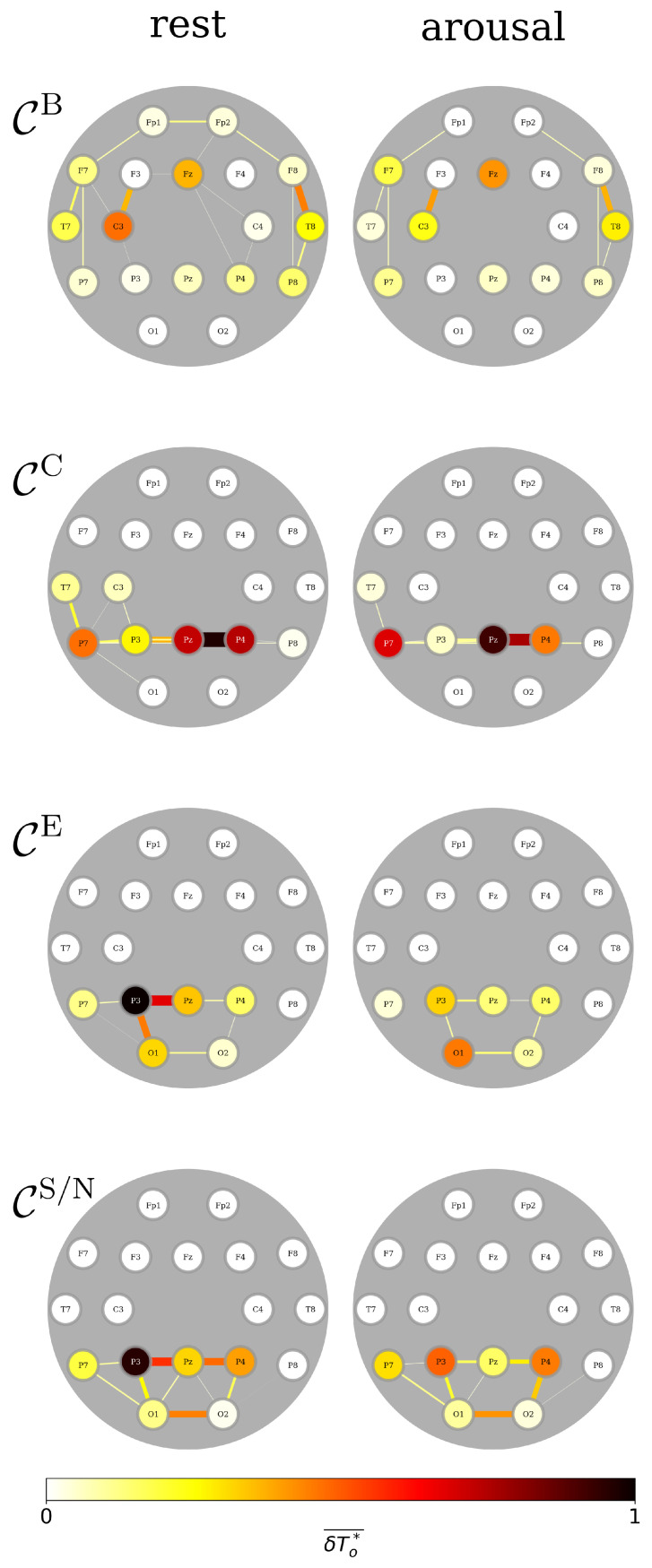
Averaged functional importance backbones (FIBs) from 30 subjects during rest and during continuous visual attention for about 30 min. See main text for group differences. Abbreviations and color-coding as in Figure A1.

A subset of 30 subjects participated a biofeedback training [44] that immediately followed the resting condition and demanded continuous visual attention for about 30 min. If we ask again with closeness centrality which FIB constituents exert most direct influence over the other constituents, we find that this task direction significantly lowered–almost 4-fold– the important role of the left precuneus (vertex P3), and the edge connecting that area with the left postcentral gyrus (vertex C3) fully lost its central role (Appendix A; Figure A6). No other FIBs and their constituents were modified by the external task direction.

### 3.4. A Day in the Life of the Brain’s FIBs: Diurnal Variations

**Figure 3:**
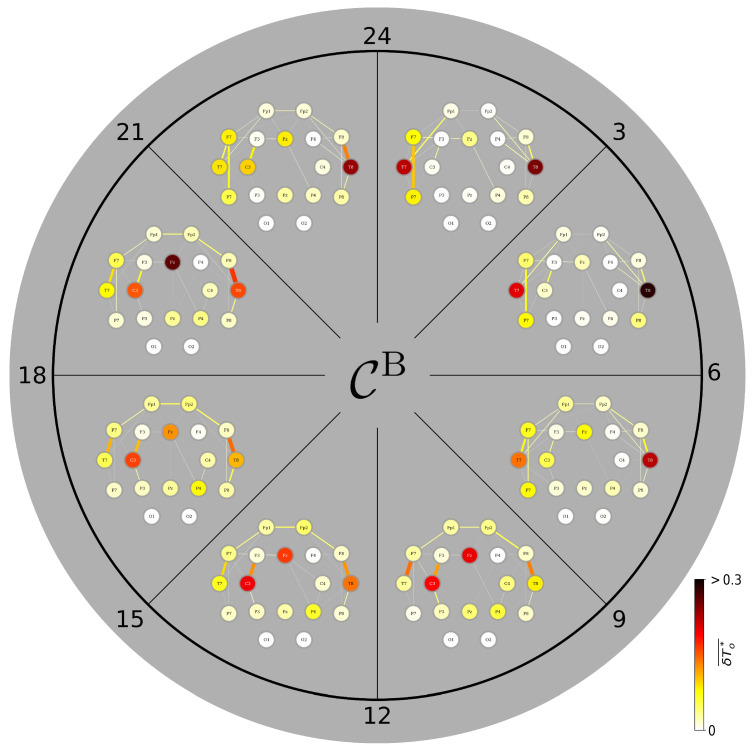
Variations of average $C^{B}$-based FIBs from nine brains during the day. Color-coded group medians of the fraction of observation time $\delta T_{o}^{★}$ during which an individual network constituent (vertex, edge) is most central. Central constituents identified with betweenness centrality $C^{B}$. Observation time is 3 h.

**Figure 4:**
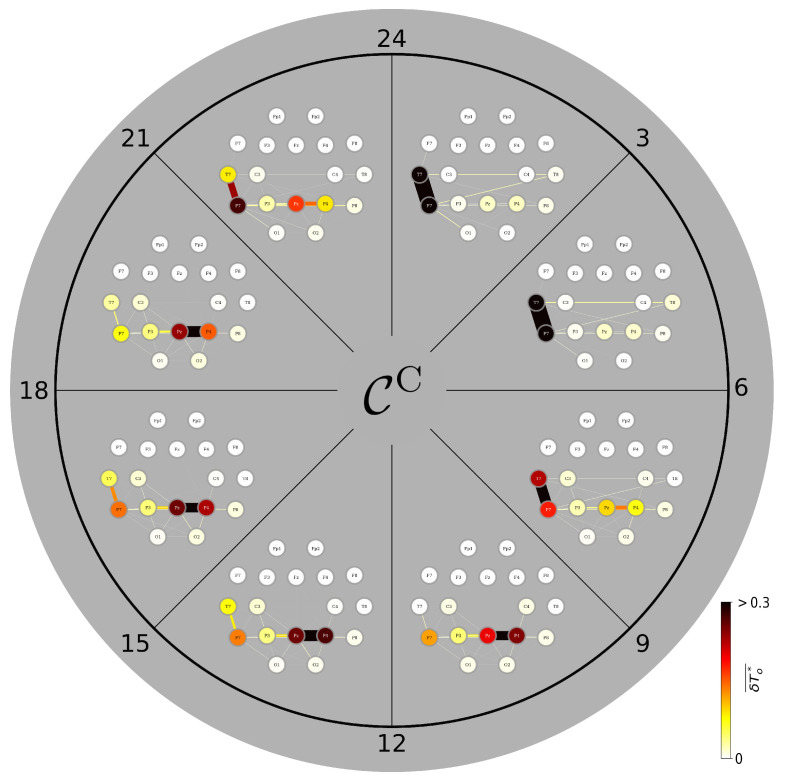
Variations of average $C^{C}$-based FIBs from nine brains during the day. Color-coded group medians of the fraction of observation time $\delta T_{o}^{★}$ during which an individual network constituent (vertex, edge) is most central. Central constituents identified with closeness centrality $C^{C}$. Observation time is 3 h.

**Figure 5:**
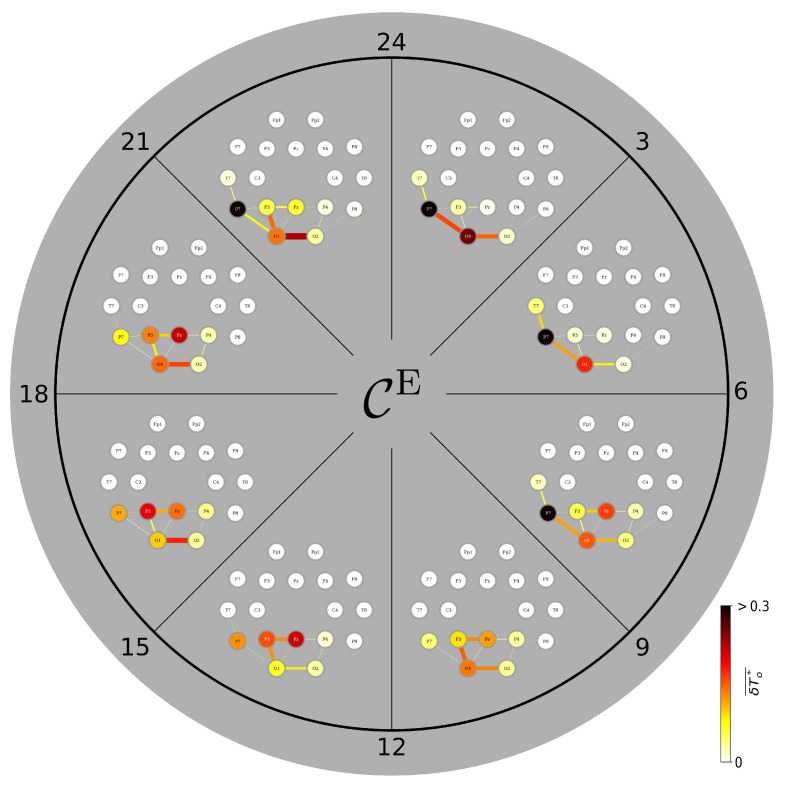
Variations of average $C^{E}$-based FIBs from nine brains during the day. Color-coded group medians of the fraction of observation time $\delta T_{o}^{★}$ during which an individual network constituent (vertex, edge) is most central. Central constituents identified with eigenvector centrality $C^{E}$. Observation time is 3 h. Note that variations of average $C^{S/N}$-based FIBs are quite similar (Appendix A; Figure A7) which underlines the spatial boundedness of the FIB that is hardly affected by sleep-wake cycle.

Given the DMN’s vital role in maintaining consciousness, awakening, emotion processing, attentional control, and working memory, its functional integration can be expected to be influenced by biological rhythms. This has indeed been observed for the circadian rhythm–a roughly 24-h cycle–using functional imaging techniques [85]. Limitations of these techniques, however, do not allow for temporally resolved studies of the influence of biological rhythms on longer timescales (days to weeks), in contrast to electrophysiological recording techniques such as the EEG [47,49]. Here, we probed for the influence of biological rhythms on the brains’ FIBs from nine subjects by performing a time-resolved (with 20 s resolution) analysis of continuous EEG recordings lasting 4–14 days. We derived individual FIBs from consecutive non-overlapping segments of 3 h duration (observation time), and collapsed the data into eight 3-h bins of a daily cycle (Figure 3, Figure 4 and Figure 5).

We start again with the focus on bottlenecks in a TEFBN’s path structure (Figure 3). During daytime (9 a.m.–9 p.m.), the corresponding FIB mainly presents with vertices and edges highlighting bilateral fronto-temporo-central brain areas and their intra- and interhemispheric interactions (cf. Figure 2) with only minor variations. Around midnight, the former, rather stable semicircular bottleneck-axis connecting the temporal lobes (vertices T7 and T8) via the frontal lobes (vertices F7, Fp1, Fp2, F8) weakens and some short-range fronto-temporal edges emerge [86]. Around the same time, the left and right middle temporal gyri (vertices T7 and T8) gain importance and retain their leading bottleneck role–with a slight left-hemispheric dominance–until the early morning. This might highlight the importance of the temporal lobes in memory consolidation during sleep [87,88].

We proceed with constituents in a TEFBN’s path structure that exert most direct influence over the other constituents (Figure 4). During daytime (9 a.m.–9 p.m.), the corresponding FIB mainly presents with the midline-right-parietal (MRP) junction (cf. Figure 2) together with vertices and edges highlighting predominantly left temporo-parietal brain areas and their short-range interactions [79,89,90,91]. This FIB and particularly its MRP junction is rather stable during the day but undergoes profound changes around midnight: the previously strong influence of the MRP junction weakens and another junction emerges that consists of the, now most influential left middle and left inferior temporal gyri (adjacent vertices T7 and P7) together with the most influential edge that connects them. This left-hemispheric junction, highlighting Wernicke’s area [92], dominates the FIB during nighttime. Interestingly, after midnight long-range interhemispheric edges emerge that connect the left-hemispheric junction with vertices highlighting the right middle and the right inferior temporal gyri. Since these connections persist until the early morning–when the sleep-related FIB reorganizes back to its awake-state counterpart –, we conjecture that this FIB highlights sleep-related memory consolidation processes [93] under the influence of the brain’s speech processing areas [94,95].

Eventually, we focus on the FIB whose constituents are most strongly integrated within a TEFBN (Figure 5). During daytime (9 a.m.–9 p.m.), the FIB present with vertices and edges predominantly highlighting left temporo-parietal and bilateral parieto-occipital brain areas (including posterior midline cortices) and their mostly short-range interactions. The only minor variations indicate a rather stable FIB, but around midnight the left inferior temporal gyrus (vertex P7) gains importance and retains its leading role as part of an emerging left temporo-occipital junction (vertices P7 and O1 together with the edge that connects them) until the early morning. We conjecture that this FIB is related to dreaming [96,97,98], to speech in dreams (or dream talking) [99,100,101], and to (verbal) memory consolidation due to the involvement of Wernicke’s area.

## 4. Discussion

Pinpointing constituents in brain-wide networks that are important for structure and function so far mainly focused on vertices that correspond to single cells, groups thereof, or even brain regions [102]. It is only recently that this vertex-centric approach has been extended by an edge-centric one [103], thereby emphasizing the role of connections and interactions for the brain’s structure and function. We go beyond these approaches and by utilizing one of the most fundamental metrics in network science—centrality—together with recent extensions jointly defined for vertices and edges, we identified functional importance backbones related to various brain dynamics ranging from wakefulness to sleep. We also go beyond the traditional approach of probing brain function via perturbations in trial-based paradigms [104,105], thereby concentrating on short-lived, mostly transient dynamics within predefined frequency bands of single vertices. Instead, we investigate how the spontaneous (ongoing) broadband dynamics of pairs of vertices shape their emergent interactions, and considering timescales from tens of seconds to days [106] that allows us to capture a wide range of (patho-)physiological activities.

Our findings highlight that resting-state brain networks are critically shaped by the dynamic interplay between vertices, in addition to their individual activities. The time-dependent edges carry complementary and, in some cases, even greater relevance in understanding the networks’ function over time. This reinforces the notion that brain function cannot be fully understood without considering the dynamic architecture of interactions (i.e, functional interaction backbones), rather than static representations of regional activity alone. This dynamic perspective helps explain why previous studies have reported only partial correspondence between resting-state networks identified with functional imaging techniques and those derived from electrophysiological data. Rather than viewing these discrepancies due to methodological differences, our findings suggest the discrepancies may reflect genuine temporal variability in how networks organize and reconfigure over longer time scales, even in the absence of explicit tasks [27,28,29,30,31,32,33]. Furthermore, our results contribute to ongoing debates about resting-state networks and their functional role [12,107]. Especially, the dynamic patterns of vertex and edge centrality we observed within brain regions associated with the default mode network suggest that the latter is not a static, monolithic structure but instead an adaptable core whose influence ebbs and flows in coordination with other brain networks.

While identifying the most important constituents of a brain network can provide valuable insights into functional importance backbones underlying specific structures and functions, it might be a concern that only these high-centrality constituents play a crucial role. Given the inherently networked nature of the brain, it is reasonable to assume that functional importance backbones emerge from the interplay of several significant, though not necessarily only the most central, vertices and edges. This issue, however, may be circumvented by taking into account different metrics that assess centrality. Other reasonable concerns are related to limitations inherent to EEG: its sensitivity to physiological and non-physiological artifacts, the notoriously ill-posed problem of choosing a suitable reference electrode, and limited brain coverage. Even though we mitigated these limitations to the best of our ability, they cannot be fully remedied. Our findings could thus be corroborated by employing other high-mobility imaging techniques that allow for continuous recordings over extended periods of time [108].

The ability to track time-varying importance of both the dynamics of brain regions and their–possibly higher-order [109,110,111]–interactions in real time over long periods of time opens exciting avenues for future research. Apart from deepening our understanding of how the brain transitions between different resting or task-related states [112], subject-specific alterations of importance may serve as biomarker for cognitive abilities or vulnerabilities to neurological disorders [113].

In summary, our work advances the study of resting-state brain networks by highlighting the dynamic and intertwined roles of brain regions and their interactions, identifying functional importance backbones. By moving beyond static descriptions and embracing changes in complex temporal brain activity on long time scales, we take a crucial step toward a richer understanding of the brain at rest, wakefulness, and sleep.

## Figures and Tables

**Table 1:** Relationship between the 10–20 standard positions and their underlying cortical structure. Abbreviations: BA = Brodmann’s area, L = left, R = right, FL = frontal lobe, TL = temporal lobe, PL = parietal lobe, OL = occipital lobe, G = gyrus.

Electrode	Hemisphere	Lobe	Anatomy	BA
Fp1	L	FL	superior frontal G	10
Fp2	R	FL	superior frontal G	10
Fz		FL	on or near interhemispheral fissure	
F3	L	FL	middle frontal G	8
F4	R	FL	middle frontal G	8
F7	L	FL	inferior frontal G	45
F8	R	FL	inferior frontal G	45
C3	L	PL	postcentral G	1,2,3
C4	R	PL	postcentral G	1,2,3
T7	L	TL	middle temporal G	21
T8	R	TL	middle temporal G	21
P3	L	PL	precuneus	19
P4	R	PL	precuneus	19
Pz		PL	on or near interhemispheral fissure	
P7	L	TL	inferior temporal G	37
P8	R	TL	inferior temporal G	37
O1	L	OL	middle occipital G	18
O2	R	OL	middle occipital G	18

**Figure A7:**
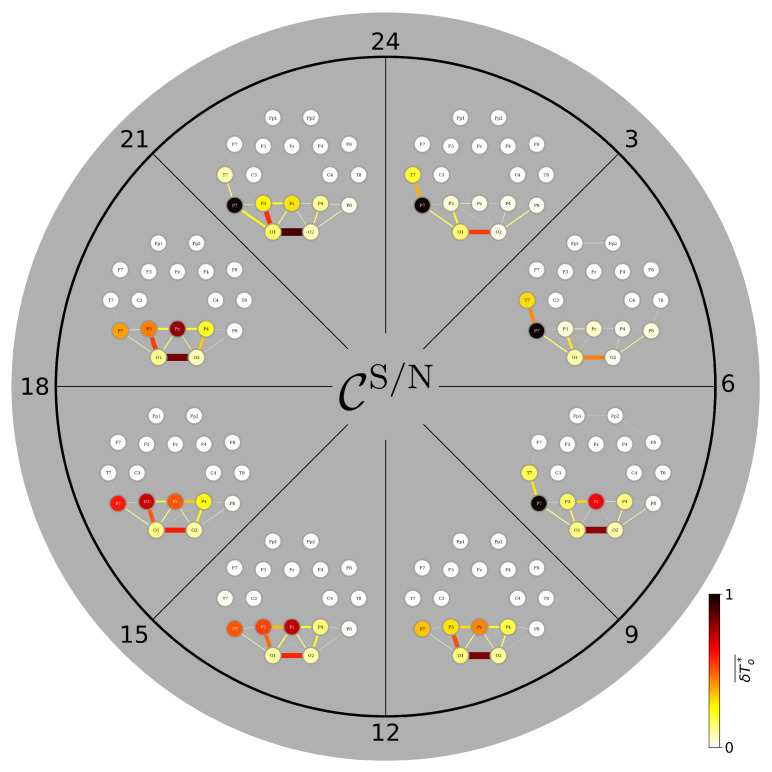
Same as Figure 5 in main text, but central constituents identified with strength/nearest neighbor centrality $C^{S/N}$.

## Data Availability

The data that support the findings of this study are available from the corresponding author upon reasonable request. The data are not publicly available as they contain information that could compromise the privacy of research participants.

## References

[1] James W., Burkhardt F., Bowers F., Skrupskelis I.K. (1890). The Principles of Psychology.

[2] Berger H. (1929). Über das Elektroenkephalogramm des Menschen. Arch. Psychiat. Nervenkrankh..

[3] Buckner R.L., Andrews-Hanna J.R., Schacter D.L. (2008). The brain’s default network: Anatomy, function, and relevance to disease. Ann. N. Y. Acad. Sci..

[4] Deco G., Jirsa V.K., McIntosh A.R. (2011). Emerging concepts for the dynamical organization of resting-state activity in the brain. Nat. Rev. Neurosci..

[5] Deco G., Corbetta M. (2011). The dynamical balance of the brain at rest. Neuroscience.

[6] Doucet G., Naveau M., Petit L., Delcroix N., Zago L., Crivello F., Jobard G., Tzourio-Mazoyer N., Mazoyer B., Mellet E. (2011). Brain activity at rest: A multiscale hierarchical functional organization. J. Neurophysiol..

[7] Cabral J., Kringelbach M.L., Deco G. (2014). Exploring the network dynamics underlying brain activity during rest. Prog. Neurobiol..

[8] Callard F., Margulies D.S. (2014). What we talk about when we talk about the default mode network. Front. Hum. Neurosci..

[9] Raichle M.E. (2015). The brain’s default mode network. Annu. Rev. Neurosci.

[10] Greicius M.D., Krasnow B., Reiss A.L., Menon V. (2003). Functional connectivity in the resting brain: A network analysis of the default mode hypothesis. Proc. Natl. Acad. Sci. USA.

[11] Uddin L.Q., Clare Kelly A., Biswal B.B., Xavier Castellanos F., Milham M.P. (2009). Functional connectivity of default mode network components: Correlation, anticorrelation, and causality. Hum. Brain Mapp..

[12] Menon V. (2023). 20 years of the default mode network: A review and synthesis. Neuron.

[13] Laufs H., Krakow K., Sterzer P., Eger E., Beyerle A., Salek-Haddadi A., Kleinschmidt A. (2003). Electroencephalographic signatures of attentional and cognitive default modes in spontaneous brain activity fluctuations at rest. Proc. Natl. Acad. Sci. USA.

[14] Mantini D., Perrucci M.G., Del Gratta C., Romani G.L., Corbetta M. (2007). Electrophysiological signatures of resting state networks in the human brain. Proc. Natl. Acad. Sci. USA.

[15] Tagliazucchi E., Von Wegner F., Morzelewski A., Brodbeck V., Laufs H. (2012). Dynamic BOLD functional connectivity in humans and its electrophysiological correlates. Front. Hum. Neurosci..

[16] Schölvinck M.L., Leopold D.A., Brookes M.J., Khader P.H. (2013). The contribution of electrophysiology to functional connectivity mapping. Neuroimage.

[17] Custo A., Van De Ville D., Wells W.M., Tomescu M.I., Brunet D., Michel C.M. (2017). Electroencephalographic resting-state networks: Source localization of microstates. Brain Connect..

[18] Kabbara A., El Falou W., Khalil M., Wendling F., Hassan M. (2017). The dynamic functional core network of the human brain at rest. Sci. Rep..

[19] Marino M., Liu Q., Samogin J., Tecchio F., Cottone C., Mantini D., Porcaro C. (2019). Neuronal dynamics enable the functional differentiation of resting state networks in the human brain. Hum. Brain Mapp..

[20] Marino M., Arcara G., Porcaro C., Mantini D. (2019). Hemodynamic correlates of electrophysiological activity in the default mode network. Front. Neurosci..

[21] De Pasquale F., Della Penna S., Snyder A.Z., Lewis C., Mantini D., Marzetti L., Belardinelli P., Ciancetta L., Pizzella V., Romani G.L. (2010). Temporal dynamics of spontaneous MEG activity in brain networks. Proc. Natl. Acad. Sci. USA.

[22] Brookes M.J., Woolrich M., Luckhoo H., Price D., Hale J.R., Stephenson M.C., Barnes G.R., Smith S.M., Morris P.G. (2011). Investigating the electrophysiological basis of resting state networks using magnetoencephalography. Proc. Natl. Acad. Sci. USA.

[23] Jin S.H., Jeong W., Lee D.S., Jeon B.S., Chung C.K. (2014). Preserved high-centrality hubs but efficient network reorganization during eyes-open state compared with eyes-closed resting state: An MEG study. J. Neurophysiol..

[24] Colclough G.L., Woolrich M.W., Tewarie P.K., Brookes M.J., Quinn A.J., Smith S.M. (2016). How reliable are MEG resting-state connectivity metrics?. NeuroImage.

[25] Duncan D., Duckrow R.B., Pincus S.M., Goncharova I., Hirsch L.J., Spencer D.D., Coifman R.R., Zaveri H.P. (2013). Intracranial EEG evaluation of relationship within a resting state network. Clin. Neurophysiol..

[26] Das A., de Los Angeles C., Menon V. (2022). Electrophysiological foundations of the human default-mode network revealed by intracranial-EEG recordings during resting-state and cognition. NeuroImage.

[27] Hutchison R.M., Womelsdorf T., Allen E.A., Bandettini P.A., Calhoun V.D., Corbetta M., Della Penna S., Duyn J.H., Glover G.H., Gonzalez-Castillo J. (2013). Dynamic functional connectivity: Promise, issues, and interpretations. NeuroImage.

[28] Baker A.P., Brookes M.J., Rezek I.A., Smith S.M., Behrens T., Smith P.J.P., Woolrich M. (2014). Fast transient networks in spontaneous human brain activity. ELIFE.

[29] Zalesky A., Fornito A., Cocchi L., Gollo L.L., Breakspear M. (2014). Time-resolved resting-state brain networks. Proc. Natl. Acad. Sci. USA.

[30] Lehnertz K., Geier C., Rings T., Stahn K. (2017). Capturing time-varying brain dynamics. EPJ Nonlin. Biomed. Phys..

[31] Lai M., Demuru M., Hillebrand A., Fraschini M. (2018). A comparison between scalp-and source-reconstructed EEG networks. Sci. Rep..

[32] Rodríguez-González V., Gómez C., Shigihara Y., Hoshi H., Revilla-Vallejo M., Hornero R., Poza J. (2020). Consistency of local activation parameters at sensor- and source-level in neural signals. J. Neural Eng..

[33] Lurie D.J., Kessler D., Bassett D.S., Betzel R.F., Breakspear M., Kheilholz S., Kucyi A., Liégeois R., Lindquist M.A., McIntosh A.R. (2020). Questions and controversies in the study of time-varying functional connectivity in resting fMRI. Netw. Neurosci..

[34] Newman M. (2018). Networks.

[35] Zuo X.N., Ehmke R., Mennes M., Imperati D., Castellanos F.X., Sporns O., Milham M.P. (2012). Network centrality in the human functional connectome. Cereb. Cortex.

[36] van den Heuvel M.P., Sporns O. (2013). Network hubs in the human brain. Trends Cogn. Sci..

[37] Power J.D., Schlaggar B.L., Lessov-Schlaggar C.N., Petersen S.E. (2013). Evidence for hubs in human functional brain networks. Neuron.

[38] De Pasquale F., Della Penna S., Sporns O., Romani G.L., Corbetta M. (2016). A dynamic core network and global efficiency in the resting human brain. Cereb. Cortex.

[39] Hadriche A., Jmail N., Blanc J.L., Pezard L. (2019). Using centrality measures to extract core pattern of brain dynamics during the resting state. Comput. Methods Programs Biomed..

[40] Lehnertz K., Ansmann G., Bialonski S., Dickten H., Geier C., Porz S. (2014). Evolving networks in the human epileptic brain. Phys. D.

[41] Bröhl T., Rings T., Pukropski J., von Wrede R., Lehnertz K. (2024). The time-evolving epileptic brain network: Concepts, definitions, accomplishments, perspectives. Front. Netw. Physiol..

[42] Bröhl T., Lehnertz K. (2019). Centrality-based identification of important edges in complex networks. Chaos.

[43] Bröhl T., Lehnertz K. (2022). A straightforward edge centrality concept derived from generalizing degree and strength. Sci. Rep..

[44] Schach S., Rings T., Bregulla M., Witt J.A., Bröhl T., Surges R., von Wrede R., Lehnertz K., Helmstaedter C. (2022). Electrodermal Activity Biofeedback Alters Evolving Functional Brain Networks in People With Epilepsy, but in a Non-specific Manner. Front. Neurosci..

[45] von Wrede R., Bröhl T., Rings T., Pukropski J., Helmstaedter C., Lehnertz K. (2022). Modifications of Functional Human Brain Networks by Transcutaneous Auricular Vagus Nerve Stimulation: Impact of Time of Day. Brain Sci..

[46] von Wrede R., Rings T., Bröhl T., Pukropski J., Schach S., Helmstaedter C., Lehnertz K. (2022). Transcutaneous Auricular Vagus Nerve Stimulation Differently Modifies Functional Brain Networks of Subjects With Different Epilepsy Types. Front. Hum. Neurosci..

[47] Bröhl T., Von Wrede R., Lehnertz K. (2023). Impact of biological rhythms on the importance hierarchy of constituents in time-dependent functional brain networks. Front. Netw. Physiol..

[48] Lehnertz H., Broehl T., Rings T., Von Wrede R., Lehnertz K. (2023). Modifying functional brain networks in focal epilepsy by manual visceral-osteopathic stimulation of the vagus nerve at the abdomen. Front. Netw. Physiol..

[49] Lehnertz K., Rings T., Bröhl T. (2021). Time in Brain: How Biological Rhythms Impact on EEG Signals and on EEG-Derived Brain Networks. Front. Netw. Physiol..

[50] Seeck M., Koessler L., Bast T., Leijten F., Michel C., Baumgartner C., He B., Beniczky S. (2017). The standardized EEG electrode array of the IFCN. Clin. Neurophysiol..

[51] Okamoto M., Dan H., Sakamoto K., Takeo K., Shimizu K., Kohno S., Oda I., Isobe S., Suzuki T., Kohyama K. (2004). Three-dimensional probabilistic anatomical cranio-cerebral correlation via the international 10–20 system oriented for transcranial functional brain mapping. Neuroimage.

[52] Koessler L., Maillard L., Benhadid A., Vignal J.P., Felblinger J., Vespignani H., Braun M. (2009). Automated cortical projection of EEG sensors: Anatomical correlation via the international 10–10 system. Neuroimage.

[53] Adamovich T., Zakharov I., Tabueva A., Malykh S. (2022). The thresholding problem and variability in the EEG graph network parameters. Sci. Rep..

[54] Varela F.J., Lachaux J.P., Rodriguez E., Martinerie J. (2001). The brain web: Phase synchronization and large-scale integration. Nat. Rev. Neurosci..

[55] Glass L. (2001). Synchronization and rhythmic processes in physiology. Nature.

[56] Uhlhaas P.J., Singer W. (2006). Neural synchrony in brain disorders: Relevance for cognitive dysfunctions and pathophysiology. Neuron.

[57] Fell J., Axmacher N. (2011). The role of phase synchronization in memory processes. Nat. Rev. Neurosci..

[58] Siegel M., Donner T.H., Engel A.K. (2012). Spectral fingerprints of large-scale neuronal interactions. Nat. Rev. Neurosci..

[59] Mormann F., Lehnertz K., David P., Elger C.E. (2000). Mean phase coherence as a measure for phase synchronization and its application to the EEG of epilepsy patients. Phys. D.

[60] O’Neill G.C., Tewarie P., Vidaurre D., Liuzzi L., Woolrich M.W., Brookes M.J. (2018). Dynamics of large-scale electrophysiological networks: A technical review. Neuroimage.

[61] Boashash B. (1992). Time Frequency Signal Analysis: Methods and Applications.

[62] Osterhage H., Mormann F., Staniek M., Lehnertz K. (2007). Measuring synchronization in the epileptic brain: A comparison of different approaches. Int. J. Bifurc. Chaos Appl. Sci. Eng..

[63] Bavelas A. (1950). Communication patterns in task-oriented groups. J. Acoust. Soc. Am..

[64] Sabidussi G. (1966). The centrality index of a graph. Psychometrika.

[65] Freeman L.C. (1979). Centrality in social networks: Conceptual clarification. Soc. Netw..

[66] Bonacich P. (1987). Power and centrality: A family of measures. Am. J. Sociol..

[67] Borgatti S.P., Everett M.G. (2006). A Graph-theoretic perspective on centrality. Soc. Netw..

[68] Freeman L.C. (1977). A Set of Measures of Centrality Based on Betweenness. Sociometry.

[69] Brandes U. (2001). A faster algorithm for betweenness centrality. J. Math. Sociol..

[70] Girvan M., Newman M.E.J. (2002). Community structure in social and biological networks. Proc. Natl. Acad. Sci. USA.

[71] Bonacich P. (1972). Factoring and weighting approaches to status scores and clique identification. J. Math. Sociol..

[72] Gonen O.M., Kwan P., O’Brien T.J., Lui E., Desmond P.M. (2020). Resting-state functional MRI of the default mode network in epilepsy. Epilepsy Behav..

[73] Esteves M., Lopes S.S., Almeida A., Sousa N., Leite-Almeida H. (2020). Unmasking the relevance of hemispheric asymmetries—Break on through (to the other side). Prog Neurobiol..

[74] Oldfield R.C. (1971). The assessment and analysis of handedness: The Edinburgh inventory. Neuropsychologia.

[75] Papadatou-Pastou M., Ntolka E., Schmitz J., Martin M., Munafò M.R., Ocklenburg S., Paracchini S. (2020). Human handedness: A meta-analysis. Psychol. Bull.

[76] Raemaekers M., Schellekens W., Petridou N., Ramsey N.F. (2018). Knowing left from right: Asymmetric functional connectivity during resting state. Brain Struct. Func..

[77] Eliot L., Ahmed A., Khan H., Patel J. (2021). Dump the “dimorphism”: Comprehensive synthesis of human brain studies reveals few male-female differences beyond size. Neurosci. Biobehav. Rev..

[78] Bluhm R.L., Osuch E.A., Lanius R.A., Boksman K., Neufeld R.W., Théberge J., Williamson P. (2008). Default mode network connectivity: Effects of age, sex, and analytic approach. Neuroreport.

[79] Sjøgård M., De Tiège X., Mary A., Peigneux P., Goldman S., Nagels G., Van Schependom J., Quinn A.J., Woolrich M.W., Wens V. (2019). Do the posterior midline cortices belong to the electrophysiological default-mode network?. Neuroimage.

[80] Vecchio F., Miraglia F., Bramanti P., Rossini P.M. (2014). Human brain networks in physiological aging: A graph theoretical analysis of cortical connectivity from EEG data. J. Alzheimer’s Dis..

[81] Petti M., Toppi J., Babiloni F., Cincotti F., Mattia D., Astolfi L. (2016). EEG resting-state brain topological reorganization as a function of age. Comput. Intell. Neurosci..

[82] Grayson D.S., Fair D.A. (2017). Development of large-scale functional networks from birth to adulthood: A guide to the neuroimaging literature. Neuroimage.

[83] Malagurski B., Liem F., Oschwald J., Mérillat S., Jäncke L. (2020). Longitudinal functional brain network reconfiguration in healthy aging. Hum. Brain Mapp..

[84] Sambataro F., Murty V.P., Callicott J.H., Tan H.Y., Das S., Weinberger D.R., Mattay V.S. (2010). Age-related alterations in default mode network: Impact on working memory performance. Neurobiol. Aging.

[85] Tian Y., Chen X., Xu D., Yu J., Lei X. (2020). Connectivity within the default mode network mediates the association between chronotype and sleep quality. J. Sleep Res..

[86] Sämann P.G., Wehrle R., Hoehn D., Spoormaker V.I., Peters H., Tully C., Holsboer F., Czisch M. (2011). Development of the brain’s default mode network from wakefulness to slow wave sleep. Cereb. Cort..

[87] Rasch B., Born J. (2013). About sleep’s role in memory. Physiol. Rev..

[88] Kaefer K., Stella F., McNaughton B.L., Battaglia F.P. (2022). Replay, the default mode network and the cascaded memory systems model. Nat. Rev. Neurosci..

[89] Culham J.C., Kanwisher N.G. (2001). Neuroimaging of cognitive functions in human parietal cortex. Curr. Opin. Neurobiol..

[90] Gottlieb J. (2007). From thought to action: The parietal cortex as a bridge between perception, action, and cognition. Neuron.

[91] De Benedictis A., Duffau H., Paradiso B., Grandi E., Balbi S., Granieri E., Colarusso E., Chioffi F., Marras C.E., Sarubbo S. (2014). Anatomo-functional study of the temporo-parieto-occipital region: Dissection, tractographic and brain mapping evidence from a neurosurgical perspective. J. Anat..

[92] Blank S.C., Scott S.K., Murphy K., Warburton E., Wise R.J. (2002). Speech production: Wernicke, Broca and beyond. Brain.

[93] Brodt S., Inostroza M., Niethard N., Born J. (2023). Sleep—A brain-state serving systems memory consolidation. Neuron.

[94] Giraud A.L., Poeppel D. (2012). Cortical oscillations and speech processing: Emerging computational principles and operations. Nat. Neurosci..

[95] Mudrik L., Deouell L.Y. (2022). Neuroscientific evidence for processing without awareness. Annu. Rev. Neurosci..

[96] Solms M. (2000). Dreaming and REM sleep are controlled by different brain mechanisms. Behav. Brain Sci..

[97] Nir Y., Tononi G. (2010). Dreaming and the brain: From phenomenology to neurophysiology. Trends Cogn. Sci..

[98] Domhoff G.W., Fox K.C. (2015). Dreaming and the default network: A review, synthesis, and counterintuitive research proposal. Conscious. Cogn..

[99] Hong C.C.H., Jin Y., Potkin S.G., Buchsbaum M.S., Wu J., Callaghan G.M., Nudleman K.L., Gillin J.C. (1996). Language in dreaming and regional EEG alpha power. Sleep.

[100] Arnulf I., Uguccioni G., Gay F., Baldayrou E., Golmard J.L., Gayraud F., Devevey A. (2017). What does the sleeping brain say? Syntax and semantics of sleep talking in healthy subjects and in parasomnia patients. Sleep.

[101] Alfonsi V., D’Atri A., Scarpelli S., Mangiaruga A., De Gennaro L. (2019). Sleep talking: A viable access to mental processes during sleep. Sleep Med. Rev..

[102] Fotiadis P., Parkes L., Davis K.A., Satterthwaite T.D., Shinohara R.T., Bassett D.S. (2024). Structure–function coupling in macroscale human brain networks. Nat. Rev. Neurosci..

[103] Betzel R.F., Faskowitz J., Sporns O. (2023). Living on the edge: Network neuroscience beyond nodes. Trends Cogn. Sci..

[104] Raichle M.E. (2015). The restless brain: How intrinsic activity organizes brain function. Philos. Trans. R. Soc. B Biol. Sci..

[105] Huk A., Bonnen K., He B.J. (2018). Beyond trial-based paradigms: Continuous behavior, ongoing neural activity, and natural stimuli. J. Neurosci..

[106] He B.J. (2014). Scale-free brain activity: Past, present, and future. Trends Cogn. Sci..

[107] Dosenbach N.U., Raichle M.E., Gordon E.M. (2025). The brain’s action-mode network. Nat. Rev. Neurosci..

[108] Stangl M., Maoz S.L., Suthana N. (2023). Mobile cognition: Imaging the human brain in the ‘real world’. Nat. Rev. Neurosci..

[109] Tudisco F., Higham D.J. (2021). Node and edge nonlinear eigenvector centrality for hypergraphs. Commun. Phys..

[110] Boccaletti S., De Lellis P., del Genio C., Alfaro-Bittner K., Criado R., Jalan S., Romance M. (2023). The structure and dynamics of networks with higher order interactions. Phys. Rep..

[111] Tabar M.R.R., Nikakhtar F., Parkavousi L., Akhshi A., Feudel U., Lehnertz K. (2024). Revealing Higher-Order Interactions in High-Dimensional Complex Systems: A Data-Driven Approach. Phys. Rev. X.

[112] Kringelbach M.L., Deco G. (2020). Brain states and transitions: Insights from computational neuroscience. Cell Rep..

[113] Badr M., Bröhl T., Dissouky N., Helmstaedter C., Lehnertz K. (2025). Stable Yet Destabilised: Towards Understanding Brain Network Dynamics in Psychogenic Disorders. J. Clin. Med..

[114] Klem G., Lüders H., Jasper H., Elger C. (1999). The international federation of clinical neurophysiology. the ten-twenty electrode system of the international federation. Electroencephalogr. Clin. Neurophysiol. Suppl..

